# Posttraumatic Aneurysm of a Patent Umbilical Vein: Diagnosis and Specific Treatment

**DOI:** 10.1055/s-0037-1604075

**Published:** 2017-07-19

**Authors:** Matthias Grade, Siegfried Krishnabhakdi, Thomas Vestring, Micha Löbermann, Joachim Conrad Arnold

**Affiliations:** 1 Department for Gastroenterology, Internal Medicine and Infectious Diseases, Christliches Krankenhaus Quakenbrück, Lower Saxony, Germany; 2 Department for Vascular Surgery, Klinikum Herford, Herford, Germany; 3 Department for Radiology, Agaplesion Diakonieklinikum Rotenburg, Rotenburg (Wümme), Germany; 4 Department for Tropical Medicine, University of Rostock, Rostock, Germany; 5 Department for Gastroenterology, Agaplesion Diakonieklinikum Rotenburg, Rotenburg (Wümme), Germany

**Keywords:** liver cirrhosis, patent paraumbilical vein, posttraumatic aneurysm, TIPS

## Abstract

A patent umbilical vein is a rare condition in healthy volunteers, but can be detected in up to 11% of patients with liver cirrhosis as a consequence of portal hypertension.

We report the case of a 52-year-old woman who was admitted to our department with acute abdominal pain after blunt trauma to her forehead and abdomen. She had a history of alcohol abuse with liver cirrhosis that had been classified as Child–Pugh stage C 5 years earlier. Signs of portosystemic shunting had been present at an earlier endoscopy, and esophageal varices were found.

Clinical examination revealed typical signs of liver cirrhosis, and ultrasound examination showed an aneurysm of 6 cm of the umbilical vein, which had not been present at earlier examinations. After lowering portal hypertension by inserting a transjugular intrahepatic portosystemic shunt, an open surgical resection of the aneurysmal umbilical vein was performed without complications. The patient recovered well and was discharged from the hospital 10 days later.

We hypothesize that the abdominal trauma prompted or aggravated umbilical vein aneurysm in this patient with liver cirrhosis and portal hypertension. Due to the risk of rupture, a surgery-based resection is a valuable treatment option.

In adults, a patent paraumbilical vein is associated with portal hypertension in liver cirrhosis, independently of the underlying cause of cirrhosis. The main etiologies of hepatic cirrhosis in Western countries are alcohol abuse, 1 2 viral hepatitis B or C, and nonalcoholic steatohepatitis. 3 In comparison to other causes of cirrhosis, alcohol abuse seems to be the most common.

A morphological enlargement of an arterial vessel is formally known as an aneurysm. The critical diameter, especially in the abdominal aorta, is estimated to be 5 cm, 4 but as other risk factors such as annual growth rate are cofactors in deterioration, these should also lead to interventional vascular procedures (operation, stenting, coils, etc.).

An enlargement of a venous vessel is described clinically as a patency, a pseudoaneurysm, or an aneurysmal dilatation. Modification in hemodynamic parameters such as increased portosystemic pressure and portal hypertension in cirrhotic liver tissue are frequently seen. 5

In addition to that, paraumbilical vein patency (PUV) is a clinical disorder with a high rate of severe complications and can found in patients with liver cirrhosis and portal hypertension.

We present the first recorded case of a woman with a PUV in liver cirrhosis following blunt abdominal trauma.

## Case Report

A 52-year-old woman with a long history of alcohol abuse presented to our hospital with trauma to her head and abdomen after domestic violence, complaining of persistent pain in her lower right abdomen. She had a history of alcoholic liver cirrhosis that had been classified as Child–Pugh stage C. In the past, she suffered from esophagus variceal bleeding that had been successfully treated by band ligation.

### Physical State

Physical examination revealed an ocular hematoma on the left eye and numerous hematomas all over the abdomen, mainly in the paraumbilical and lower regions. There were clinical signs of ascites. Body weight was 68 kg, height was 168 cm, and body mass index was 24. There were no specific neurologic findings and no signs of hepatic encephalopathy.

### Laboratory Results on Admission

Routine laboratory tests revealed normal full blood count. Platelets were 31/nL (normal range: 150–350/nL), and hemoglobin was 8.2 g/dL (normal range: 12–16 g/dL).

Sodium 128 was mmol/L (normal range: 135–145 mmol/L), potassium was 3 mmol/L (normal range: 3.5–4.5 mmol/L), and calcium was 2.09 mmol /L (normal range: 2.1–2.5 mmol/l).

Signs of decreased liver function due to liver cirrhosis were detected: bilirubin was 6.90 mg/dL (normal range: <1.2), GOT (AST) was 69 U/L (normal range: <32), GPT (ALT) was 33 U/L (normal range: <33), GGT was 532 U/L (normal range: 6–42), choline esterase was 3.18 kU/L (normal range: 5.3–12.9). LDH was 355 U/L (normal range: <250), C-reactive protein was 1.2 mg/dL (normal range: <0.5 mg/L).

Liver coagulation parameters: INR was 1.53(normal range 1–1.5), and PTT was 35.6 second (normal range: 24–33 seconds).

### Physical Examinations and Interventional Procedures

**Fig. 1:**
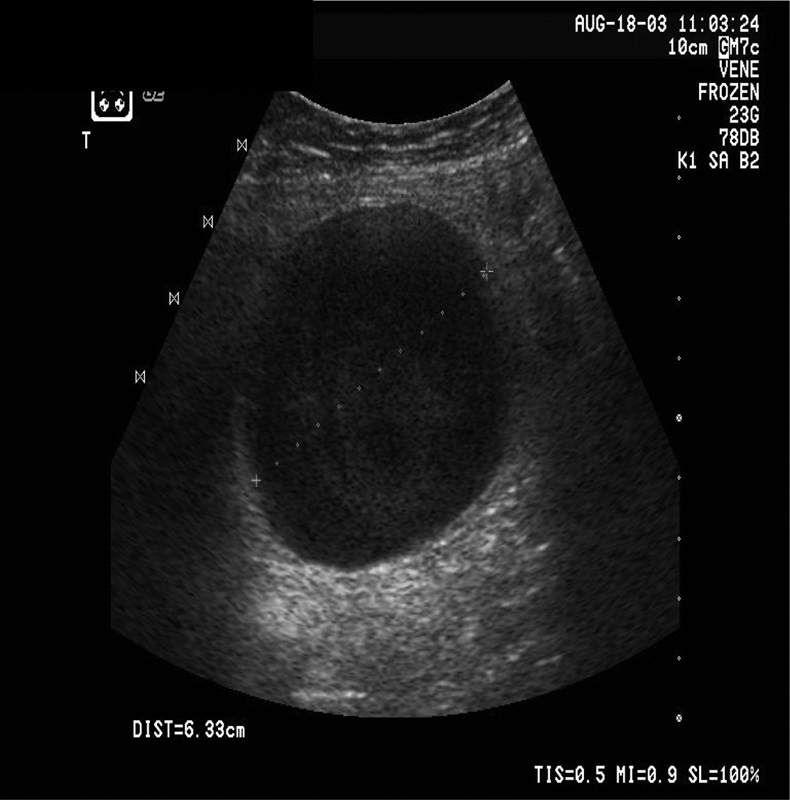
Under ultrasound examination, an echo-poor formation can be identified as a vessel.

**Fig. 2:**
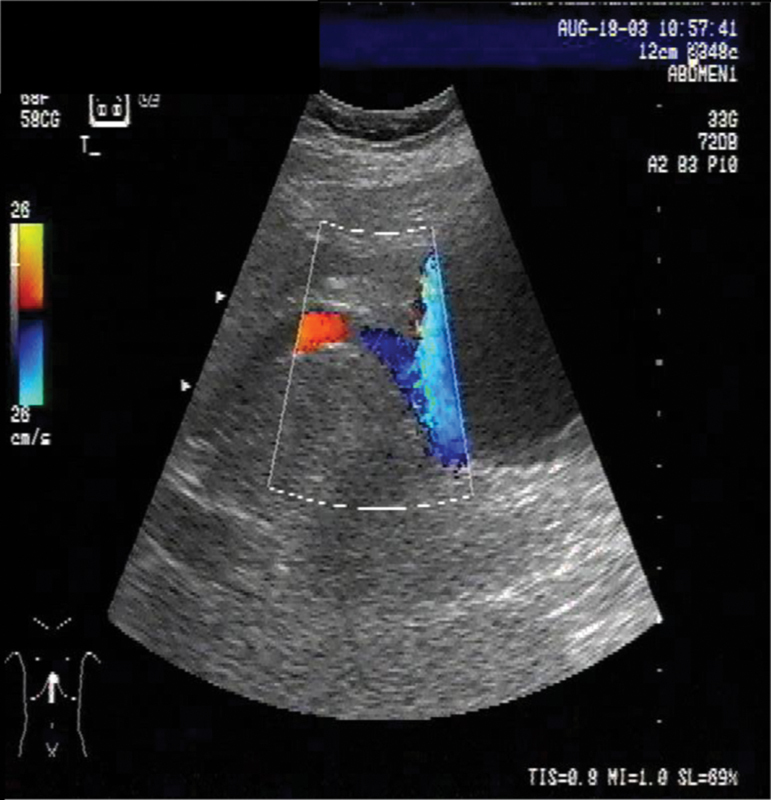
In the power Doppler mode, the superficial abdominal varices are clearly visible.

An abdominal ultrasound examination immediately after hospital admission detected liver cirrhosis with a moderate amount of ascites (normal light yellow ascites with no signs of spontaneous bacterial peritonitis with neutrophil count < 250/mm ^3^ ), and an enlarged vessel with aneurysm-like ballottement over an area of 5 to 6 cm near the paraumbilical region. The vessel was identified as the patent umbilical vein ( Figs. 1 and 2 ).

**Fig. 3:**
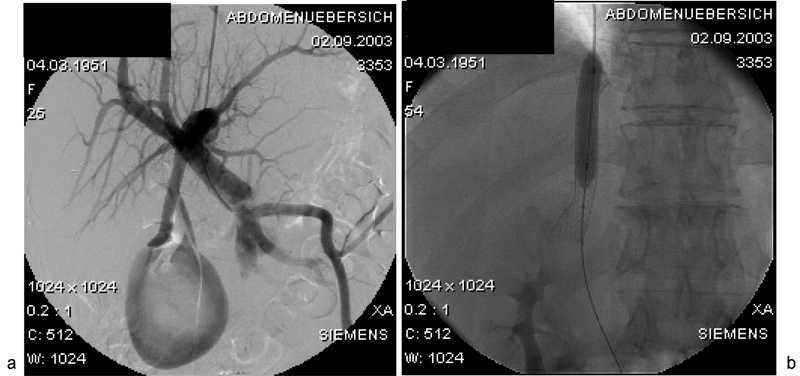
( **a** ) Transjugular intrahepatic portosystemic shunt (TIPS) ( **b** ) Due to the presence of esophageal varices and a patent umbilical vein, a TIPS was implanted before surgical correction commenced to reduce the portovenous pressure and to minimize the risk of intraoperative bleeding.

Treatment of the aneurysm was indicated due to the patient's unstable social environment, which put her at risk of recurrent blunt traumata and potentially fatal bleeding. Voluminous abdominal wall collaterals made a laparoscopic approach inappropriate. As a result, a team of gastroenterologists, surgeons, and interventional radiologists met to discuss the most appropriate procedure. In the light of the patient's elevated bilirubin levels (6.9 mg/dL), and fearing the spontaneous rupture of the aneurysm, the decision was taken to lower portal pressure. The implantation of a transjugular intrahepatic portosystemic shunt (TIPS; Fig. 3a, b ) seemed to be the least invasive option. Apart from thrombocytopenia and elevated bilirubin, contraindications for TIPS implantation (hepatic encephalopathy, portal thrombosis, heart insufficiency, pulmonal hypertension, etc.) could be ruled out.

### Transjugular Intrahepatic Portosystemic Shunt Procedure

The TIPS maneuver was performed in a standard fashion through the right jugular vein. Following intrahepatic portal vein puncture, the direct portography revealed a 5.8 × 7 cm large aneurysm of the patent umbilical vein. For TIPS creation, the parenchymal tract was dilated using a 7-mm angioplasty balloon followed by placement of a 12-mm bare stent (Memotherm-TIPS, BARD GmbH, Karlsruhe, Germany). Depending on the portosystemic gradient, balloon dilation of the stent was successively performed from 7 to 9 mm. Following 9-mm angioplasty, the portosystemic pressure gradient dropped from 24 mm Hg before the procedure to 13 mm Hg after the procedure.

**Fig. 4:**
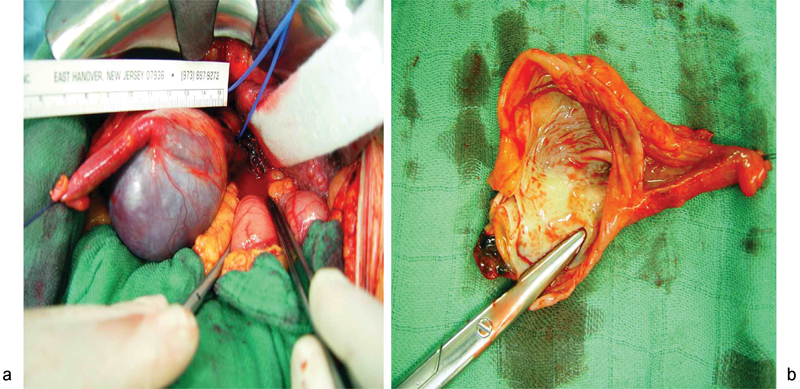
Intraoperative findings. ( **a** ) The paraumbilical vein patency is ligated but still in situ. ( **b** ) Typical combined saccular and fusiform-type aneurysm.

Due to persisting aneurysm after TIPS procedure visible in ultrasound examination, and regarding the patient's social background and the volatility of her personal situation after hospital discharge, a complete surgical removal of the aneurysm was suggested as the best, most reliable solution ( Fig. 4a , b ).

### Surgical Procedure

During the operation, the aneurysm was exposed by a midline incision ( Fig. 4a ), and the large abdominal wall vessels were ligated. The patent umbilical vein was resected from the umbilicus to the interlobular level close to the portal vein ( Fig. 4b ). Unusual adhesions to the falciform ligament and the gallbladder, combined with the thinness of the aneurysm wall, made preparation extensive and demanding and encouraged the theory of traumatic origin.

The resected vessel was incised longitudinally, and the aneurysm was identified as a combined saccular and fusiform type ( Fig. 4b ). The abdominal cavity was closed without drainage after meticulous bleeding control. Overall blood loss was minimal.

The patient was discharged 10 days after operation in good condition and put on a withdrawal and rehabilitation program.

## Discussion

This clinical case focuses on the controversial question of interventional procedures in cases of aneurysm of the umbilical vein. A review of the literature indicates that a *posttraumatic* origin of an umbilical vein aneurysm has never been described to date. Although we have no direct proof that the physical violence exerted by the patient's partner actually caused the aneurysmatic enlargement to develop, but the theory seems to be credible. A histopathology of the resected aneurysm was not proposed unfortunately. The last laparoscopic examination performed 2 years earlier in our endoscopic unit showed no signs of aneurysm. The port of the laparoscopic device was placed at Kalk's point in the left mid-abdomen at that time, clarifying the cirrhotic liver situation.

To avoid a spontaneous rupture, for example, a rupture during resection, our aim was to reduce portovenous pressure to lower levels.

The patient already had spontaneous bleeding from esophageal varices. After discussion with the patient and assurances of her intention to make changes in her personal life (agreement to embark on an alcohol withdrawal and rehabilitation program), we took the decision to perform a vascular surgical correction of the aneurysm.

Due to the high risk of intraoperative bleeding complications, a TIPS was implanted before surgery. TIPS implantation in cirrhotic patients started in 1988 as an alternative to surgery-based shunting procedures. Nowadays, TIPS is an established form of treatment that has been shown to increase survival in patients with end-stage liver cirrhosis. 6 7 8

In conclusion, this rare case demonstrates the impact of violent acts to the special liver tissue system in cirrhotic alcohol-addicted patients.

The patient ultimately recovered well. At the time of discharge, she was free of ascites and was able to embark on her chosen withdrawal therapy.

Outlook and patient-related procedure: after withdrawal therapy, endoscopic checks for esophageal varices and ultrasound examinations of the TIPS will be performed on a regular basis. The cirrhotic situation will be kept under strict control in the outpatient department of the liver unit.

## References

[1] Chen C H Wang J H Lu S N Comparison of prevalence for paraumbilical vein patency in patients with viral and alcoholic liver cirrhosis Am J Gastroenterol 2002 97 09 2415 2418 12358266 10.1111/j.1572-0241.2002.05996.x

[2] Zardi E M Uwechie V Caccavo D Portosystemic shunts in a large cohort of patients with liver cirrhosis: detection rate and clinical relevance J Gastroenterol 2009 44 01 76 83 19159076 10.1007/s00535-008-2279-1

[3] Altinbas A Sowa J P Hasenberg T Canbay A The diagnosis and treatment of non-alcoholic fatty liver disease Minerva Gastroenterol Dietol 2015 61 03 159 169 26080905

[4] Chang J B Stein T A Liu J P Dunn M E Risk factors associated with rapid growth of small abdominal aortic aneurysms Surgery 1997 121 02 117 122 9037221 10.1016/s0039-6060(97)90279-8

[5] Jalan R Hayes P C British Society of Gastroenterology. UK guidelines on the management of variceal haemorrhage in cirrhotic patients Gut 2000 46(Suppl 3-4):III1 III15 10862604 10.1136/gut.46.suppl_3.iii1 PMC1766736

[6] European Association for the Study of the Liver.EASL clinical practice guidelines on the management of ascites, spontaneous bacterial peritonitis, and hepatorenal syndrome in cirrhosis J Hepatol 2010 53 03 397 417 20633946 10.1016/j.jhep.2010.05.004

[7] Fujimoto J Gene therapy for liver cirrhosis J Gastroenterol Hepatol 2000 15(Suppl):D33 D36 10759218 10.1046/j.1440-1746.2000.02146.x

[8] Hara Y Sato Y Yamamoto S Successful laparoscopic division of a patent ductus venosus: report of a case Surg Today 2013 43 04 434 438 22945888 10.1007/s00595-012-0316-4

